# Effects of continuous infusion of etomidate at various dose rates on adrenal function in dogs

**DOI:** 10.1186/s12871-015-0171-0

**Published:** 2016-01-08

**Authors:** Bangyong Qin, Hongzhuan Hu, Baofeng Cao, Zhaoqiong Zhu

**Affiliations:** Department of Anesthesiology, Affliated Hospital of Zunyi Medical College, No. 149 Dalian Road, Zunyi, Guizhou Province 563003 China

**Keywords:** Intravenous anesthesia, Etomidate, Adrenal function

## Abstract

**Background:**

Etomidate is a commonly used sedative in intravenous anesthesia. The aim of this study was to compare the effects of various etomidate doses administered by continuous infusion on adrenal function in dogs under general anesthesia.

**Methods:**

Thirty-six healthy adult male dogs were randomly divided into six groups. Sodium pentobarbital alone was administered to the control group (group C); five experimental groups (E_1_, E_2_, E_3_, E_4_, and E_5_) were also given etomidate at doses of 10, 15, 20, 25, and 30 μg · kg^−1^ · min^−1^, respectively, to maintain anesthesia. Heart rate (HR), mean arterial pressure (MAP), and bispectral index (BIS) were monitored. Serum cortisol, aldosterone, adrenaline, and noradrenaline levels were measured, and HR, MAP, and BIS values recorded, before intubation (T_0_), and at 1 h, 2 h, and 3 h after intubation (T_1–3_).

**Results:**

Cortisol and aldosterone levels in groups E_1–5_ decreased as the doses and times of continuous infusion of etomidate increased. The cortisol level was significantly decreased compared with baseline at T_3_ in group E_1_ and at T_1–3_ in groups E_2–5_ (*P* < 0.05). Compared with the corresponding levels in group C, cortisol levels were significantly lower than T_0_ values at T_3_ in group E_1_ and at T_1–3_ in groups E_2–5_ (*P* < 0.05). The aldosterone level was significantly lower at T_3_ in group E_2_ and at T_1–3_ in groups E_3–5_ (*P* < 0.05). Significant reductions in cortisol levels at T_2–3_ in group E_2_ and at T_1–3_ in groups E_3–5_ compared with group C were also observed (*P* < 0.05). The plasma adrenaline and noradrenaline levels, HR, MAP, and BIS in groups E_1–5_ were within the normal range at the different times and with the different doses (*P* > 0.05).

**Conclusions:**

Cortisol and aldosterone levels decreased with time and continuous infusion of etomidate; there were no significant changes in adrenaline and noradrenaline levels, HR, MAP, and BIS in any group.

## Background

Etomidate is a short-acting sedative with rapid onset of effects that is commonly used for inducing short-term anesthesia prior to intubation in patients. Advantages of this general anesthetic agent include rapid recovery of consciousness and minimal influence on tidal volume and systemic pressure. It is safe and especially suitable for patients with coronary heart disease, hypertension, old age, or shock [1, 2]. Etomidate is an intravenous induction agent that is associated with hemodynamic stability following administration [3, 4]. Compared with propofol, etomidate shows greater hemodynamic stability after induction of anesthesia, and may be preferred over propofol for general anesthesia [5]. Etomidate should be considered for induction of anesthesia in cardiac surgery patients [5, 6]. One of the most common—but important—side effects of this drug is the suppression of steroid production by the reversible inhibition of the 11-beta-hydroxylase enzyme [3]. Etomidate is rarely used to maintain anesthesia because of this adrenal suppression [7]. However, some studies have reported that when administered to induce anesthesia, the suppression is transient, and others have noted no significant inhibitory effect on adrenocortical function in patients with severe sepsis or septic shock [8–10]. The pros and cons of etomidate continue to be discussed, and its use remains controversial [11–13]. The objective of this study was to evaluate the effects of continuous infusion of various doses of etomidate on adrenal function in dogs. Therefore, applications and disadvantages of etomidate relying on disputed, especially whether they have a significant effect on adrenal function. Our research aims to provide reference for clinical application.

## Methods

### Animals and groups

All procedures were conducted in accordance with the rules and regulations of the Subcommittee on Research Animal Care at Zunyi Medical College. Thirty-six healthy adult male mongrel dogs of the same breed, eight to 12 months of age, and weighing 10 ± 2.5 kg were used for the study. The dogs were fasted 12 h prior to the experiment and were randomly divided into six groups. Dogs in the control group (group C) were anesthetized with sodium pentobarbital only. Dogs in experimental groups (E_1_, E_2_, E_3_, E_4_, and E_5_) underwent induction of anesthesia and were intubated using sodium pentobarbital and then maintained under anesthesia for 3 h with a continuous infusion of etomidate at doses of 10, 15, 20, 25, and 30 μg · kg^−1^ · min^−1^, respectively. This study was carried out in strict accordance with the recommendations in the Guide for the Care and Use of Laboratory Animals of the National Institutes of Health. The animal use protocol has been reviewed and approved by the Institutional Animal Care and Use Committee (IACUC) of Zunyi Medical College.

### Medicines and instruments

Etomidate was obtained from Xuzhou Enhua Pharmaceutical Co., Ltd. (batch number: 20100430), and vecuronium bromide (it is no histamine release and little effect on hemodynamics and cheap [14]) was obtained from Zhejiang Xianju Pharmaceutical Co., Ltd. (batch number: 100503). An RY-R type anesthesia machine was purchased from Jiangsu Kaitai Medical Equipment Co., Ltd. A multi-function monitor (UT4000Fpro) was purchased from Shenzhen Kaitai Industry Co., Ltd., and UP8000 depth of anesthesia monitors were purchased from the Shenzhen Kerui Kang Industrial Co., Ltd. The manufacturers is GC-2010γRadio immunity counter, University of Science and Technology of China science and Technology Industrial Corporation.

### Anesthetic methods

The dogs were pre-treated with 2.5 % sodium pentobarbital (25,000 μg/kg) intraperitoneally at 8:15 to 8:30 on the procedure day, and catheters were placed in the right femoral artery and vein. Sodium heparin (3000 μg/kg) was infused via a central venous line, and mean arterial pressure (MAP), heart rate (HR), and bispectral index (BIS) were monitored. Animals were heparinized to facilitate the collection of blood samples. Group C animals were anesthetized with 2.5 % sodium pentobarbital (500 μg/kg) and supplemented at appropriate intervals by intraperitoneal injection to maintain a BIS value of 40–60 (it is depth of anesthesia and close clinical anesthesia state). In addition to sodium pentobarbital, dogs in the five experimental groups (E_1_, E_2_, E_3_, E, and E_5_) received infusions of etomidate at doses of 10, 15, 20, 25, and 30 μg · kg^−1^ · min^−1^, respectively, and were maintained under anesthesia for 3 h. Respiratory parameters were set as follows: tidal volume, 15 mL/kg; respiratory rate, 15–18 times per minute; I:E, 1:2; oxygen flow rate, 2 L/min; and P_ET_CO_2_, 4.66-5.99 kPa. Each dog received 4–6 mL · kg^−1^ · h^−1^ Lactated Ringer’s solution IV to maintain the central venous pressure (CVP) between 6 and 10 cmH_2_O. Subsequently intermittent boluses of vecuronium bromide (50 μg/kg) were administered for neuromuscular blockade to maintain the muscle relaxant. The temperature of the controlled laboratory was 23 °C and the animals’ temperature was 37–38 °C.

### Observation indexes

Blood samples were collected from the right femoral vein at time points T_0_ to T_3_ for radioimmunoassay analysis of serum cortisol, aldosterone, adrenaline, and noradrenaline concentrations [15]. MAP, HR, and BIS were recorded at the same time points. Indeed, the half-life of serum cortisol, serum aldosterone and ACTH in human blood is 70 min [16], 20 min [17], and 10 min [18], respectively.

### Statistical analysis

All data are reported as the mean ± SD. Statistical analyses were performed using SPSS17.0 software. Single factor analysis of variance was compared between groups. Pair-wise comparisons underwent post hoc testing with Dunnett’s *t* test. For all statistical analyses, *P* < 0.05 indicated statistical significance.

## Results

### Cortisol and aldosterone levels

**Fig. 1:**
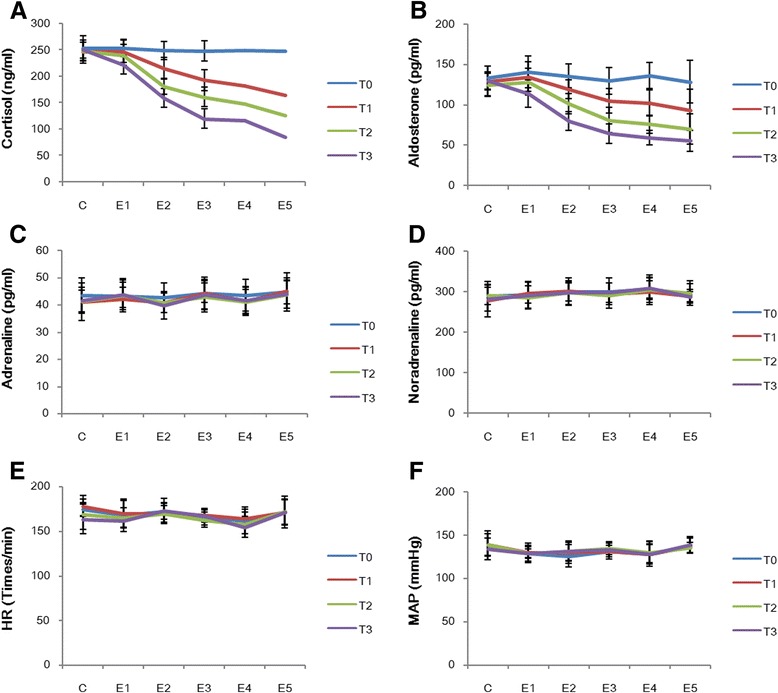
Changes of several indexes with different doses (10, 15, 20, 25, and 30 μg•kg^−1^•min^−1^) at different time points (T_0_, T_1_, T_2_ and T_3_). **a** Changes of cortisol with different concentrations in different groups; (**b**) Changes of aldosterone with different concentrations in different groups; (**c**) Changes of epinephrine with different concentrations in different groups; (**d**) Changes of noradrenaline with different concentrations in different groups; (**e**) Changes of heart rate with different concentrations in different groups; (**f**) Changes of mean arterial pressure with different concentrations in different groups

Results are summarized in Table 1 (Fig. 1). The cortisol and aldosterone levels in groups E_1–5_ decreased as the dose of etomidate and length of infusion time increased. Compared with T_0_ values, the cortisol level was significantly decreased at T_3_ in group E_1_ and at T_1–3_ in groups E_2–5_ (*P* < 0.05). For example, the level was 215.40 ± 19.51 in group E_2_ significantly lower than the corresponding level (246.76 ± 16.95) in group C. Compared with group C, cortisol was significantly decreased relative to T_0_ values at T_3_ in group E_1_ and at T_1–3_ in groups E_2–5_ (*P* < 0.05). The aldosterone level was significantly decreased at T_3_ in group E_2_ and at T_1–3_ in groups E_3–5_ (*P* < 0.05); in comparison with the corresponding values in group C, the aldosterone levels were significantly lower at T_2–3_in group E_2_ and at T_1–3_ in groups E_3–5_ (*P* < 0.05). Compared with the values in group C, serum cortisol and aldosterone concentrations in groups E_1_-E_5_ demonstrated a dose-dependent reduction (*P* < 0.05) (Table 1 and Fig. 1).

**Table 1:** Six group dogs cortisol, aldosterone concentration change and comparison $\left(\mathrm{n}=6,\;\overline{x}\pm \mathrm{s}\right)$

Index	Group	T_0_	T_1_	T_2_	T_3_
Cortisol (ng/ml)	C	253.73 ± 23.87	246.76 ± 16.95	247.88 ± 16.81	251.53 ± 18.62
	E1	254.10 ± 22.10	247.70 ± 21.24	240.95 ± 20.71	222.72 ± 19.87*^#^
	E2	249.25 ± 17.15	215.40 ± 19.51*^#^	181.32 ± 14.89*^#^	159.63 ± 18.47*^#^
	E3	248.43 ± 16.99	193.33 ± 17.95*^#^	161.31 ± 17.43*^#^	120.14 ± 18.05*^#^
	E4	249.72 ± 20.41	182.80 ± 19.14*^#^	148.80 ± 17.42*^#^	116.33 ± 10.84*^#^
	E5	248.28 ± 23.02	165.32 ± 20.56*^#^	126.43 ± 16.82*^#^	85.69 ± 12.90*^#^
Aldosterone (pg/ml)	C	133.93 ± 14.37	129.15 ± 18.78	124.89 ± 14.00	129.83 ± 11.23
	E1	140.54 ± 19.63	134.77 ± 18.35	128.48 ± 16.74	113.81 ± 16.67
	E2	135.76 ± 15.01	119.28 ± 12.01	100.98 ± 12.65^#^	79.70 ± 11.66^*#^
	E3	129.61 ± 16.51	105.24 ± 15.30^*#^	80.98 ± 15.80^*#^	64.28 ± 11.88^*#^
	E4	136.29 ± 16.52	102.07 ± 16.28^*#^	75.95 ± 11.34^*#^	59.37 ± 8.98^*#^
	E5	128.55 ± 26.43	93.66 ± 25.79^*#^	69.87 ± 18.70^*#^	55.35 ± 13.18^*#^

And T_0_ comparison, *P < 0.05; and C group comparison, ^#^ *P* < 0.05

### Adrenaline and noradrenaline levels

Results are summarized in Table 2 (Fig. 1). The plasma adrenaline and noradrenaline levels in groups E_1–5_ were within the normal range at the different times and doses (*P* > 0.05).

**Table 2:** The plasma epinephrine, norepinephrine concentration and HR, MAP, BIS value in six group dogs $\left(\mathrm{n}=6,\;\overline{x}\pm \mathrm{s}\right)$

Index	Group	T_0_	T_1_	T_2_	T_3_
Adrenaline (pg/ml)	C	43.53 ± 6.24	41.09 ± 6.91	41.29 ± 4.04	41.66 ± 4.87
	E1	43.32 ± 5.85	42.28 ± 4.22	43.61 ± 4.73	43.87 ± 5.70
	E2	42.65 ± 5.39	40.73 ± 3.68	40.87 ± 3.68	39.82 ± 5.02
	E3	44.29 ± 5.77	44.37 ± 3.64	43.05 ± 5.72	43.87 ± 5.56
	E4	43.50 ± 5.89	41.49 ± 5.31	41.05 ± 4.71	41.66 ± 4.72
	E5	44.73 ± 7.10	45.08 ± 4.89	43.73 ± 4.98	43.96 ± 5.26
Noradrenaline (pg/ml)	C	288.86 ± 36.37	277.90 ± 40.30	291.46 ± 23.99	281.36 ± 29.27
	E1	292.10 ± 32.97	297.13 ± 25.21	285.51 ± 29.30	291.17 ± 32.93
	E2	300.00 ± 34.08	301.12 ± 24.32	298.77 ± 22.61	297.21 ± 30.30
	E3	300.43 ± 32.74	295.03 ± 26.47	290.53 ± 31.35	297.63 ± 24.39
	E4	306.56 ± 33.67	300.71 ± 33.29	305.37 ± 21.80	307.97 ± 27.67
	E5	292.54 ± 27.16	288.37 ± 16.86	296.69 ± 30.30	287.81 ± 12.12
HR (Times/min)	C	174.50 ± 7.48	178.17 ± 11.6	169.00 ± 16.82	163.33 ± 16.53
	E1	168.00 ± 18.17	169.50 ± 14.99	165.00 ± 11.49	162.00 ± 7.54
	E2	172.33 ± 9.25	169.50 ± 9.14	170.00 ± 11.45	172.67 ± 13.71
	E3	165.33 ± 8.21	168.33 ± 6.83	162.83 ± 8.38	167.67 ± 7.42
	E4	161.83 ± 15.15	164.33 ± 9.95	157.33 ± 14.15	154.67 ± 12.00
	E5	171.00 ± 13.76	171.00 ± 17.82	172.00 ± 14.30	171.33 ± 14.68
MAP (mmHg)	C	138.00 ± 16.57	138.67 ± 12.79	139.00 ± 12.77	134.17 ± 12.70
	E1	129.33 ± 11.04	129.83 ± 6.74	128.83 ± 9.17	128.83 ± 5.04
	E2	125.83 ± 12.86	129.83 ± 12.92	131.50 ± 11.31	131.17 ± 10.42
	E3	131.50 ± 9.48	131.83 ± 6.68	134.67 ± 7.23	133.33 ± 8.31
	E4	128.33 ± 11.22	128.50 ± 14.36	130.17 ± 12.35	127.83 ± 11.39
	E5	138.50 ± 8.34	137.67 ± 9.00	135.50 ± 5.86	139.00 ± 9.14
BIS	C	47.67 ± 3.33	47.50 ± 1.76	48.17 ± 2.93	46.83 ± 3.66
	E1	46.50 ± 5.05	47.50 ± 4.76	46.50 ± 4.59	48.17 ± 5.49
	E2	43.83 ± 3.06	45.83 ± 4.12	44.50 ± 2.81	45.00 ± 3.10
	E3	47.33 ± 3.72	46.67 ± 3.39	49.33 ± 3.39	49.83 ± 5.04
	E4	48.00 ± 2.61	45.17 ± 3.66	46.83 ± 3.06	43.83 ± 2.48
	E5	45.33 ± 4.10	44.83 ± 2.86	44.17 ± 3.13	44.00 ± 2.45

### Hemodynamics and BIS

Results are summarized in Table 2 (Fig. 1). HR, MAP, and BIS values in groups E_1–5_ were within the normal range at the different times and doses (*P* > 0.05).

## Discussion

Etomidate was introduced into clinical practice in 1972, and initial reports of its use in humans emerged in the clinical literature soon afterward [19]. Etomidate is the only imidazole among the general anesthesia induction drugs, and it has the most favorable therapeutic index for single bolus administration [4, 6]. Etomidate induces less apnea than barbiturates or propofol, does not cause histamine release, and very rarely causes allergic reactions [5, 20]. Because of its remarkably benign hemodynamic effects, etomidate has proven useful for general anesthetic induction in patients undergoing cardiac surgery and those with poor cardiac function [21, 22]. The major molecular targets mediating the anesthetic effects of etomidate in the central nervous system are specific γ-aminobutyric acid type A receptor subtypes. It also produces a unique toxicity among anesthetic drugs: inhibition of adrenal steroid synthesis that far outlasts its hypnotic action [20]. Adrenal cortical inhibition by etomidate has received a great deal of attention and significantly limits its use as both an anesthetic and a sedative [23, 24]. A vigorous debate regarding the use of etomidate for intubation continues [25, 26].

The adrenal cortex secretes cortisol and aldosterone, and their concentrations may reflect adrenocortical function. Because cortisol has a circadian rhythm, we induced anesthesia at the same time every day during our study. We designed our experiments which references for the clinical dose range 10–20 μg · kg^−1^ · min^−1^of etomidate and dog doses equivalent to the human 1.88 times [20]. The cortisol and aldosterone levels in groups E_1–5_ demonstrated time- and dose-dependent reductions with the continuous infusion of etomidate. Our results therefore show that intravenous infusion of etomidate can inhibit adrenal cortical functions. The adrenal medulla secretes epinephrine and norepinephrine. Catecholamines are important indicators of the stress response and have obvious correlations with hemodynamic changes. In our study, plasma epinephrine and norepinephrine concentrations stayed within normal ranges in the experimental dogs and there were no statistically significant differences at the various times in the control group. HR and MAP remained within normal ranges as etomidate doses and times increased. Etomidate anesthesia does not have a significant effect on perioperative epinephrine and norepinephrine levels. Etomidate, used as a continuous infusion at 10–30 μg · kg^−1^ · min^−1^ for colonoscopy can maintain a good depth of anesthesia and results in faster recovery than propofol-remifentanil sedation [27]. Etomidate also can maintain good clinical anesthesia depth (BIS value) as the dose increases. In our study, BIS values showed minimal change with different doses of etomidate, probably because of the lack of surgical stimulation and the use of a muscle relaxant. Meanwhile, experiments were carried out under isothermal conditions which did not affect the results. Although the altered temperature may affect the depth of anesthesia, we carefully monitored the temperature of dogs during the experiment.

Our study used continuous infusion of different doses of etomidate in dogs and determined that serum cortisol and aldosterone levels were decreased as the time and doses of etomidate infusion increased, but there were no significant changes in adrenaline, noradrenaline, or hemodynamic values. As etomidate administered at 10 μg · kg^−1^ · min^−1^ for 3 h had minimal effect on adrenocortical and adrenal medulla function, use of etomidate at this dose may be safe for anesthesia maintenance.

We did not perform ACTH stimulation tests in this experiment, mainly because pre-anesthetic testing in the study dogs revealed normal adrenal function. However, this is a limitation of our experiment.

## Conclusion

In summary, the study showed that cortisol and aldosterone levels decreased with time and continuous infusion of various etomidate in dogs under general anesthesia; there were no significant changes in adrenaline and noradrenaline levels, HR, MAP, and BIS in any group. These results provide reference for clinical application.

## Abbreviations

HR: Heart rate
MAP: Mean arterial pressure
BIS: Bispectral index
ICU: Intensive care unit
CVP: Central venous pressure
